# Enhancing Antimicrobial Activity of Thyme Essential Oil Through Cellulose Nano Crystals-Stabilized Pickering Emulsions

**DOI:** 10.3390/foods13223706

**Published:** 2024-11-20

**Authors:** Andreas Romulo, Vania Salsabila Anjani, Ata Aditya Wardana

**Affiliations:** Food Technology Department, Faculty of Engineering, Bina Nusantara University, Jakarta 11480, Indonesia; vania.anjani@binus.ac.id (V.S.A.); ata.wardana@binus.ac.id (A.A.W.)

**Keywords:** antimicrobial, CNC, essential oil, Pickering emulsion

## Abstract

Essential oils (EOs), such as thyme essential oil (TEO), are widely known for their antimicrobial properties; however, their direct application in food systems is limited due to their poor stability, which affects their efficacy. This study aims to improve the stability and antimicrobial efficacy of TEO by encapsulating it in Pickering emulsions stabilized with cellulose nanocrystals (CNC). Two formulations of Pickering emulsions with 5% and 10% TEO were prepared and compared to traditional surfactant-based emulsions. The stability of the emulsions was assessed over 21 days, and particle size, zeta potential, Raman spectroscopy, and FTIR were used for characterization. The antimicrobial activity was tested against several foodborne pathogens, with minimum inhibitory concentration (MIC) values determined. The 10% TEO Pickering emulsion showed antimicrobial activity, with MIC_50_ values of 4096 µg/mL against *Staphylococcus aureus* and *Escherichia coli*, while the 5% TEO formulation had no effect at MIC_50_ > 8192 µg/mL. The CNC-stabilized Pickering emulsions exhibited superior stability, showing no phase separation over 21 days. The findings suggest that CNC-stabilized Pickering emulsions are effective at improving the stability and antimicrobial performance of TEO, making them a promising natural preservative for food packaging and safety. Further research is recommended to optimize the formulation and broaden TEO’s application in food preservation.

## 1. Introduction

Food is undeniably vital for life, making food safety a priority for both consumers and the food industry [1]. However, as food is an agricultural product, food spoilage is a significant challenge in the food industry, as it leads to substantial economic losses and can compromise food safety [2]. Consumption of spoiled food, mainly due to the presence of pathogenic microorganisms, causes food-related diseases. According to the World Health Organization (WHO), each year, 1 in 10 people suffer from foodborne illnesses, resulting in approximately around 420,000 deaths [3]. Therefore, to tackle this problem, food must be preserved to maintain its quality over an extended period. Food preservation refers to the methods or techniques used to control internal and external factors that can lead to food spoilage. The main goal of these preservation processes is to extend the shelf life of food while preserving its original nutrients, color, texture, and taste [4].

In the food and agricultural industries, synthetic preservatives are commonly used to maintain product quality. Available options include sodium benzoate, sodium propionate, potassium sorbate, sorbic acid, nitrites, sulfites, nisin, and natamycin, along with potassium lactate, ascorbic acid, citric acid, and tartaric acid [5]. These have all been approved by regulatory authorities for keeping the quality and safety of food products [6]. However, in recent years, the use of synthetic chemicals to control pathogens in food has raised significant concerns regarding human health [7]. Certain synthetic antimicrobial agents, even those approved by regulatory bodies for use as food preservatives, pose potential health risks to consumers [8,9]. As a result, there has been a growing shift toward using natural and safer alternatives, which appeal to consumers seeking more “green” or organic options. Among these alternatives, plant essential oils (EOs) have garnered considerable interest for their effectiveness as natural food preservatives, either applied directly to food or incorporated as packaging substances [10,11].

EOs, also known as volatile aromatic oils, are natural, complex liquids with distinct aromas and flavors that vary depending on their specific chemical composition [12]. Produced by aromatic plants as secondary metabolites, these oils are especially prevalent in species from warmer regions, like tropical and Mediterranean climates, where they have long been used in traditional medicine. The production of EOs in plants is intended to attract pollinators or repel predators, while their chemical components act as phytohormones, serving as signaling molecules and growth regulators [13]. These biological properties lend EOs antimicrobial and antioxidant functionalities, and they have been utilized in food preservation since ancient times [5,14]. EO_S_ contain over 50 compounds, mainly terpenes like monoterpenoids and sesquiterpenoids, along with aromatic compounds (phenylpropanoids, aldehydes, alcohols, esters) and aliphatic compounds (alkanes, aldehydes, alcohols, ketones, esters) [15]. In some EOs, a single compound may constitute over 20% of the composition [16]. Currently, more than 3000 essential oils have been identified, with around 300 having commercial value in industries such as food, pharmaceuticals, sanitation, and cosmetics. For example, thyme (*Thymus vulgaris* L., Lamiaceae) essential oil is widely used in food and cosmetics and is recognized for its antibacterial, antifungal, anticancer, anti-inflammatory, and antiviral properties [17]. Despite the demonstrated antimicrobial effectiveness of essential oils (EOs), their full potential in food systems is restricted by challenges such as poor bioavailability, water solubility, stability, and volatility [18,19]. When exposed to light, heat, oxygen, or interactions with food matrices, EO components can undergo changes that reduce their antioxidant and antimicrobial capabilities [20]. To address these issues, EOs can be encapsulated through emulsification. Emulsion systems are widely used in the food industry to modify texture, flavor, and appearance in products like milk, cream, mayonnaise, beverages, and sauces. This process involves liquid–liquid dispersions of two immiscible substances, where one is dispersed as droplets within the other. Emulsifiers are often added to enhance stability by surrounding the dispersed droplets, reducing interfacial tension, or increasing droplet-to-droplet repulsion [21]. Among emulsion techniques, Pickering emulsions (PEs) have gained attention due to their unique stabilization by solid particles rather than traditional surfactants, offering enhanced stability, reduced health risks, and an eco-friendly alternative using natural, food-safe particles [22].

Cellulose nanocrystals (CNC) are particularly effective as Pickering particles due to their lightweight structure, large surface area, high crystallinity, amphiphilic properties, minimal toxicity, and versatility [23]. CNC has been recognized to improve mechanical properties of coatings and films in food packaging [24]. In terms of direct application in food, CNC has been used as stabilizer to prolong the shelf life of frozen product such as ice cream [25], improving the overall quality of ice cream, while at the same time maintaining the sensory acceptability [26]. Although CNCs are considered to have minimal to undetectable antimicrobial effects [27], surface modification with antimicrobial agents has been used as a strategy to develop CNC-based hybrid materials with enhanced antimicrobial properties [28]. Additionally, CNCs play an important role in delivery systems [29], particularly in encapsulating bioactive compounds of essential oils within Pickering emulsion systems [30,31,32,33].

Understanding the characteristics of PEs, along with testing their biological activity, is crucial for their use in food applications. To the best of the authors’ knowledge, there is a gap in comprehensive studies on the antimicrobial activity of PE–TEO stabilized by CNC particles. Based on this, the present research aimed to characterize PE–TEO stabilized by CNC and evaluate its antimicrobial activity against several pathogenic microorganisms responsible for food-borne illnesses.

## 2. Materials and Methods

### 2.1. Materials

TEO was obtained from Essential Formula (Indonesia). Some chemicals used for analysis such as Mueller Hinton Broth (MH-Broth) (Merck, Darmstadt, Germany), Tween 80 (Merck, Germany), sodium alginate (Shandong Jiejing Corp., Rizhao, China), and CNC powder (Nippon Paper Industries Co., Ltd., Tokyo, Japan).

### 2.2. Microorganisms and Grwoth Media Preparation

In this study, six bacterial strains and two fungal strains were tested. The following American Type Culture Collection (ATCC) and NITE Biological Resource Center (NBRC) standard strains were used: *Staphylococcus aureus* ATCC 29213, *Staphylococcus aureus* ATCC 25923, *Staphylococcus aureus* NBRC 13276, *Bacillus subtilis* NBRC 3009, *Bacillus cereus* IFO 3001, *Escherichia coli* NBRC 13500, *Candida albicans* ATCC 14053, and *Penicillium digitatum* NBRC 7758. These microorganism cultures were maintained in Mueller-Hinton broth (MHB) (Merck, Germany) at 4 °C until use. MHB equilibrated with Tris-buffered saline (Merck, Jakarta, Indonesia) served as the culture medium. For inoculum standardization [34], the turbidity of the microorganism suspensions was adjusted to a 0.5 McFarland standard (1.5 × 10^8^ CFU/mL) using a DEN-1B McFarland densitometer (Biosan, Riga, Latvia).

### 2.3. GC-MS Analysis

The identification of the chemical composition of EO was performed following a previous method [35] with modification. The composition of TEO was analyzed using a Gas Chromatography-Mass Spectrometry (GC-MS) system 7890B equipped with mass selective detector (MSD) 5977A and a HP-5MS column (30 m × 250 µm × 0.25 µm) (Agilent Technologies, Santa Clara, CA, USA). Around 1 µL of sample was injected with a 5:1 split ratio injection, using helium (He) as the carrier gas at a flow rate of 1 mL/min, with an initial oven temperature of 40 °C ramping at 10 °C/min to 300 °C (held for 4 min), an injector temperature of 250 °C, a transfer line temperature of 270 °C, and mass spectrometric detection in scan mode (30–600 *m*/*z*) using electron ionization (EI) at 70 eV, where the MS source and quadrupole were set at 230 °C and 150 °C, respectively, and data acquisition began after a 4 min solvent delay to ensure accurate identification of essential oil components. The composition of thyme essential oil was identified based on its retention time and comparison of mass spectra from NIST library.

### 2.4. Preparation of Sodium Alginate-Pickering Emulsion Thyme Essential Oil

Sodium alginate and PE–TEO were prepared according to the previous methodology described with modification [30]. A 1% solution of Na-alginate was prepared by dissolving the powder into distilled water with proper mixing at 700 rpm using a magnetic stirrer. Subsequently, the CNC powder was prepared by dissolving in distilled water to prepare 0.2% of CNC solution. Then, 5% and 10% of PE–TEO was prepared by mixing TEO to 0.2% of CNC solution and 1% of Na-alginate solution, homogenized using T25 digital ultra-turrax homogenizer (IKA-Werke GmBH,, Staufen, Germany)) at 15,000 rpm for 3 min. Afterward, the PE solution was degassed by putting it in a vacuum chamber. For making the film, the PE–TEO solution was cast on silicon plates and dried at 35 °C for 24 h. The film was then removed from the cast, packaged into aluminum foil, and stored in a desiccator filled with saturated magnesium nitrate (MgNO_3_)_2_. For comparison, Tween 80-based emulsion of TEO was prepared by dissolving 1% Tween 80, TEO, and Na-alginate, following the same procedure.

### 2.5. Antimicrobial Activity Evaluation

The antimicrobial activity of TEO and PE–TEO was assessed using a modified broth microdilution technique for determining the Minimum Inhibitory Concentration (MIC) [35], which refers to the lowest concentration of an antimicrobial agent that prevents microbial growth after incubation. In this procedure, 100 μL of buffered MH-Broth solution was placed into each well of a microplate. Following this, 200 μL of TEO that was dissolved in 1% DMSO and PE–TEO that was dissolved in the buffered broth and were introduced into a 96-well microplate. Serial two-fold dilutions were then carried out, beginning with an initial concentration of 8192 μg/mL. Afterward, 5 μL of bacterial suspension, standardized to a turbidity equivalent to 0.5 McFarland (1.5 × 10^8^ CFU/mL), was added to each well. The positive control contained only bacteria without the samples, while the negative control included only the broth without bacteria. The microplates were incubated at 37 °C for 24 h, and absorbance readings were taken at 405 nm using a Biotek Epoch microplate reader (Agilent Technologies, Santa Clara, CA, USA). Each test was performed in triplicate to determine the MIC for each sample, and the method was applied to both PE solution samples and emulsions. The results were expressed as MIC_80_ and MIC_50_, which represented the minimum concentration that must be achieved for 80% and 50% of inhibition. Tetracycline and tioconazole (Merck KGaA, Darmstadt, Germany) were used as positive control.

### 2.6. Stability Test

Each coating solution of PE–TEO and TEO emulsion was stored at room temperature for 21 days to assess its stability. The stability of the emulsions was evaluated by visually inspecting their appearance in photographs taken over the storage period. Photographs were taken with a Samsung Galaxy A54 smartphone under sufficient lighting (Resolution: 4080 × 3060 pixels, 12 MP; ISO: 200; Focal Length: 23 mm; Aperture: f/1.8; Shutter Speed: 1/40 s).

### 2.7. Particle Size Analysis and Zeta Potential Analysis

The particle size analysis of the samples was conducted using a Malvern Panalytical ZS XPLORER (Malvern Panalytical, Malvern, UK) utilizing dynamic light scattering (DLS) under steady-state conditions. The sample was dispersed in water, with the dispersant having a refractive index (RI) of 1.33, viscosity of 0.887 cP, and a dielectric constant of 78.5, while the temperature was maintained at 25 °C. The particle size distribution was measured by intensity, volume, and number, with the material’s refractive index set to 1.52 and an absorption value of 0.01.

### 2.8. Raman Spectroscopy Analysis

Raman spectroscopy analysis of the Pickering emulsion was conducted using a DXR3xi Raman Imaging Microscope (Thermo Fischer Scientific, Waltham, MA, USA). The sample was placed in a holder to prevent spillage, and the instrument was set to irradiate the sample with a laser at 5 mW power, using 1000 scans, with an exposure time of 0.001 s and a frequency of 100 Hz. The DXR3xi model offers fine laser power control in 0.1 mW increments, with a wavelength of 532 nm. The microscope has a spatial resolution of 0.5 µm and a confocal depth of 2 µm, ensuring precise imaging and spectral capture. The spectrometer was calibrated to detect Raman shifts in the range of ~500 cm^−1^ to ~3000 cm^−1^, capturing characteristic peaks corresponding to various molecular interactions within the sample.

### 2.9. Fourier-Transfor Infra Red (FTIR) Analysis

The FTIR analysis was conducted using a Nicolet iS10 FTIR Spectrometer equipped with an ATR Diamond accessory (Thermo Fischer Scientific, Waltham, MA, USA). Organic samples were first prepared by tearing the film of samples into smaller pieces to facilitate the measurement of the chemical composition. The spectra for each sample were recorded within the wavenumber range of 400–4000 cm^−1^, capturing key functional group information.

## 3. Results and Discussion

### 3.1. GC-MS Analysis of Thyme Essential Oil (TEO)

The chemical composition of thyme essential oil in this study has been identified and summarized in Table 1. About 58 compounds were detected, representing 96.32% of the total compounds. The TEO analyzed contains monoterpenes, monoterpenoids, sesquiterpenes, and some miscellaneous or unclassified compounds. Most of the oil is composed of monoterpenoids, which account for 49.42% of the total composition, followed by monoterpenes, which contribute 25.66% to the oil’s composition. Sesquiterpenes account for 4.75% of the total, representing a smaller fraction of the oil. Miscellaneous or unclassified compounds make up 0.72%, and unidentified compounds contribute approximately 1.57%.

The major compound detected in TEO is thymol (29.10%), followed by p-cymene (14.80%), carvacrol (7.46%), gamma-terpinene (6.08%), endo-borneol (4.13%), and eucalyptol (3.74%). In previous research, thymol has also been identified as the major compound of thyme essential oil, accounting for 17.4% [36], 47.59% [37], 60.55% [38] of the total oil. Thymol, a compound derived from thyme (*Thymus vulgaris*) essential oil, is a monoterpenoid produced through the hydroxylation of p-cymene, following the aromatization of γ-terpinene into p-cymene [39]. Thymol as the dominant compound detected has been reported as an active ingredient responsible for biological activities of TEO [17]. p-cymene, a monocyclic monoterpene hydrocarbon, is known as precursor for synthesizing thymol and carvacrol [40]. As a single compound, p-cymene possessed numerous pharmacological properties, including antimicrobial, antioxidant, anti-inflammatory, antidiabetic, anti-tumor, and anticancer [40,41,42,43,44]. Likewise with thymol, carvacrol, an isomeric monoterpenoid of thymol, has been reported for its used as preservative and therapeutic effect for medicinal purposes [45]. The amount of carvacrol present in the thyme essential oil (TEO) in this study was lower than that of thymol, which is consistent with findings from previous studies.

The disparity between the chemical composition of TEO observed in this study and in previous studies could be attributed to several internal and external factors. Internal factors within the plant could include its genetic characteristics (such as species, ecotype, and chemotype), population density, origin, specific plant parts, growth stage, seasonal timing of sampling, as well as physiological and biochemical pathways, with the plant’s overall physiology, the development stage of synthesizing tissues, and metabolic functions playing particularly important roles [46]. In addition, external factors include a range of environmental influences, such as climate, habitat conditions, soil composition, geographic location, as well as the timing and methods of harvest [47]. For instance, two distinct chemotypes of Catalan TEO, 1,8-cineole and linalool, have been identified in samples collected from various altitudes [48]. Chbel et al. [49] studied the impact of geography on TEO from Morocco and France. They found that Moroccan oils had high levels of borneol, α-terpinol, and carvacrol (31.04%, 15.16%, and 7.13%, respectively), while French oils contained more thymol, ortho-cymene, and γ-terpinene (35.77%, 17.23%, and 8.05%, respectively. Similarly, the variation in the chemical composition of TEO from Iran was also reported by Nezhadali et al. [49], with different stages of plant growth resulting in varying TEO yields (0.83–1.39%) and levels of thymol (38.23–63.01%) and o-cymene (5.56–15.47%). It should also be noted that different postharvest handling techniques may result in isolation of certain compounds while excluding others, therefore influencing the final composition of essential oil [50].

### 3.2. Stability of Pickering Emulsion Thyme Essential Oil (PE–TEO)

The stability of the emulsion is one of the most important characteristics to factor in when evaluating the performance of an emulsion, as an unstable emulsion can lead to phase separation, sedimentation, and degradation of components, which have negative impacts on the products. In this research, we created two different formulas for the emulsion of TEO, namely Pickering emulsion and surfactant emulsion, and measured their particle size, zeta potential, and polydispersity index (Table 2).

Generally, PE–TEO (Sample A and B) have larger particle sizes compared to the surfactant emulsions (Sample C and D). PE–TEO 5% has a larger particle size (8917 nm) compared to PE–TEO 10% (4994 nm). Similar conditions were also observed for the surfactant emulsions, for which sample C gives a larger particle size (1682) than sample D (619 nm). The findings in this research contrast with the previous study that reported that the increase in essential oil concentration could increase the particle size of the emulsion [51]. The phenomenon observed in this study is probably due to the idea that emulsifier molecules might rearrange more efficiently at the oil-water interface, forming a more structured or denser stabilizing layer. Tween 80-stabilized emulsions (Samples C and D) result in much smaller particle sizes compared to Pickering-stabilized emulsions (Samples A and B), which correspond to the previous study that revealed the same phenomenon [52]. Cellulose nanocrystals (CNCs), as solid particles, stabilize emulsions by creating a physical barrier around oil droplets, which is probably less effective in reducing surface tension compared with Tween 80. As a result, CNC-stabilized emulsions tend to have larger particles [53].

Zeta potential is primarily used to assess the electrical characteristics of droplets in nanoemulsions because it provides a clear representation of their electric properties and is easy to calculate [54]. Emulsion systems with zeta potential that value higher than ±30 mV are considered to have a good physical stability, minimize the occurrence of flocculation, and prevent coalescence [55]. Based on Table 1, both Sample C (−19.17 mV) and Sample D (−13.34 mV) have much lower zeta potential values than Sample A (−40.29 mV) and Sample B (−38.91 mV). Moreover, sample C and D have a relative high polydispersity index, accounting for 0.5548 and 1.0000, respectively. Compared to sample A (0.3522) and sample B (0.2562) that indicated a more uniform particle size distribution, emulsion formulated with Tween 80 has the highest degree of heterogeneity particle size. Despite its lower particle size, the use of Tween 80 alters the stabilization from electrostatic forces to steric mechanism [56].

**Figure 1:**
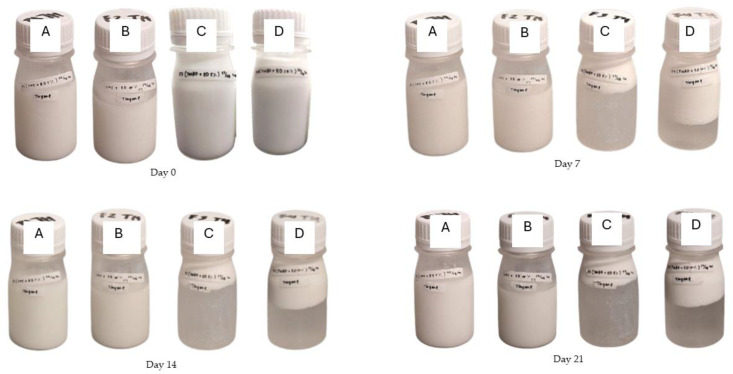
Emulsion stability test of PE–TEO and Tween 80-TEO. (**A**): CNC 0.2% + TEO 5% + Na-alginate 1%; (**B**): CNC 0.2% + TEO 10% + Na-alginate 1%; (**C**): Tween 80 1% + TEO 5% + Na-alginate; (**D**): Tween 80 1%, TEO 10%, Na-alginate.

The results are consistent with the stability test conducted. The stability of the emulsion was observed over 21 days, comparing the PE–TEO with a Tween 80-stabilized TEO. As shown in Figure 1, no physical differences were observed between the formulated samples on day 0, indicating a well-formed and stable emulsion. Up to 21 days, Samples A and B maintained stability without any signs of separation. However, Samples C and D showed noticeable phase separation. The visual data corresponded with the measured zeta potential and PDI values, which indicate that Samples C and D had a zeta potential greater than −30 mV and a higher PDI, suggesting that they were less stable compared to Samples A and B, as evidenced by the phase separation.

The Pickering emulsion showed enhanced stability due to the amphiphilic nature of CNC, which adsorbs at the oil–water interface, minimizing the interfacial tension between the oil and water phases [57]. Increasing the concentration of TEO in the PE led to a slight decrease in the zeta potential value of PE–TEO, from −40.29 mV to −38.91 mV. Although there is no specific report on the influence of varying TEO concentrations on the zeta potential of CNC-stabilized Pickering emulsions, a study has reported a decrease in zeta potential values in nanoemulsions with higher EO concentrations [58]. Interestingly, other research has indicated that increasing the EO concentration can either decrease or increase the zeta potential value [59]. Therefore, it is likely that the zeta potential value is more influenced by the emulsifier used. In this study, the slight reduction in zeta potential did not negatively impact stability, as the zeta potential of PE–TEO 10% remained below −30 mV, indicating strong electrostatic repulsion between droplets, preventing the coalescence, that contributed to the observed emulsion stability [60]. On the other hand, the emulsion stabilized by Tween 80 is categorized as having incipient instability, as indicated by zeta potential values of −19.17 mV and −13.34 mV, which fall within the ±10 mV to ±30 mV range [61]. This instability led to phase separation at 7, 14, and 21 days of storage. Our results align with previous research showing that the Pickering Emulsion (PE) technique produces more stable emulsions than those formulated with Tween 80, as Tween 80 emulsions showed separation after only 1 day of storage [62]. The zeta potential of Tween 80-stabilized TEO approaches zero, contributing to emulsion instability observed in this study, likely due to its non-ionic surfactant nature and environmental stress during emulsion production [63].

### 3.3. Raman Spectroscopy and FTIR Results

**Figure 2:**
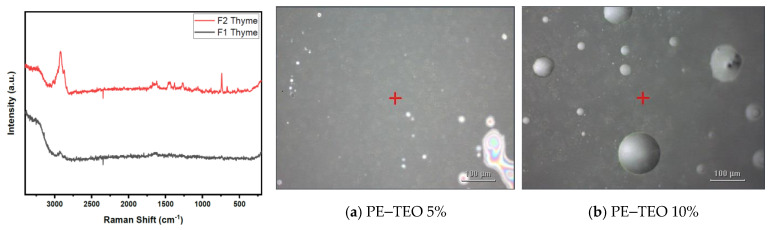
Raman shift and optical image of Pickering emulsion (**a**) TEO 5%, (**b**) TEO 10%.

Figure 2 illustrates a comparison between two Pickering emulsions (PE) containing thyme essential oil (TEO) at varying concentrations. The black line represents PE–TEO 5% (F1), while the red line depicts PE–TEO 10% (F2). The spectrum for PE–TEO 10% shows greater intensity compared to PE–TEO 5%, suggesting a stronger signal and likely a higher concentration of active components contributing to the Raman signals. Prominent peaks between 3000 cm^−1^ and 500 cm^−1^ correspond to molecular vibrations from the thyme essential oil and the emulsion matrix. The most significant peaks, which appear with greater intensity in F2, are likely associated with C-H, C=C, and other functional groups in the essential oil.

The key peaks identified include the C-COO stretch at approximately 700–800 cm^−1^, the C-C stretch around 1000–1100 cm^−1^, and the CH_2_ twist at 1200–1300 cm^−1^. Additionally, a notable C=C stretch was observed at about 1600 cm^−1^, along with C-H vibrations near 3000 cm^−1^. This character aligns with previous findings where four major essential oil components from oregano and thyme using FT-Raman spectroscopy were identified in the spectral region from 1800 to 600 cm^−1^ [64]. These findings confirm the effective incorporation of thyme essential oil into the Pickering emulsion and suggest that molecular interactions depend on the concentration. Higher concentrations lead to more distinct peaks and increased spectral intensity, indicating that the emulsion structure becomes more defined as the thyme essential oil concentration rises, shedding light on the system’s stability and composition. The increased intensity observed in the spectrum of PE–TEO 10% (F2) compared to PE–TEO 5% (F1) aligns with findings from previous research, which also reported that higher concentrations of essential oils lead to stronger Raman signals [65].

**Figure 3:**
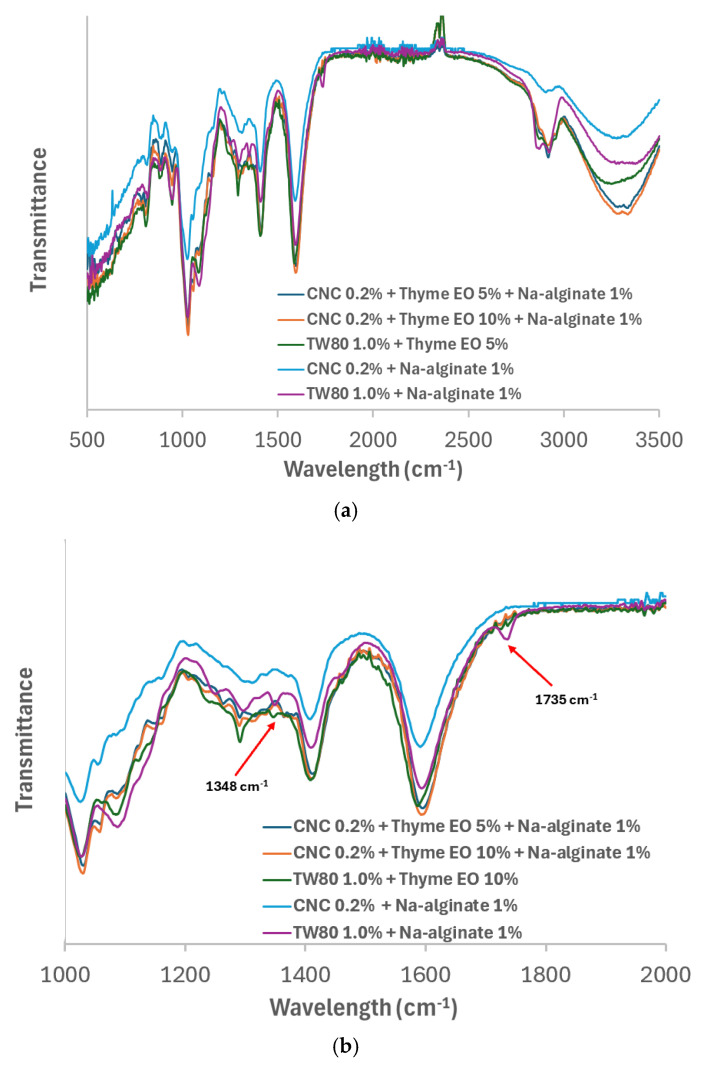
FTIR spectra of Pickering emulsion. (**a**) full-spectrum analysis (500–3500 cm^−1^); (**b**) expanded spectrum (1000–2000 cm^−1^).

The chemical structure and interactions between the materials were analyzed using ATR-FTIR to evaluate the characteristic absorption bands of the film coating. The vibrational spectra at specific wavelengths revealed the presence of chemical bonds [66]. As shown in Figure 3a, the ATR-FTIR spectra of all samples exhibit a broad absorption band (3200–3300 cm^−1^), which corresponds to the O–H bond stretching involved in the intermolecular interactions between alginate monomers. This band also indicates the formation of an emulsion, where compounds like Tween 80 and CNF bind to water in the solution, leading to the formation of hydrogen bonds between water and the binding compounds [67]. The previous study reported that the broad band observed at 3267 cm^−1^ in the alginate coating sample corresponded to the stretching vibrations of O–H groups [68]. The intensity of this band increased progressively with the addition of essential oil, suggesting enhanced hydrogen bonding interactions between these additives and the hydroxyl groups within the film [68]. However, this study found different results: a decrease in intensity was observed when nanocellulose was added to Na-alginate, suggesting that hydrogen bonding interactions between the alginate matrix and CNC may disrupt the molecular network between β-d-mannuronic acid and α-l-guluronic acid blocks.

A similar effect was seen for the absorption bands at 1348 cm^−1^ and 1735 cm^−1^ (marked with a red arrow in Figure 3b), which were only significantly observed in the sample containing 1.0% TW80 + 1% Na-alginate. These bands correspond to the stretching of CO and C–C bonds in alginate and were absent when the Pickering emulsion was emulsified. Moreover, the intensity of the absorption band at 3200–3300 cm^−1^ increased when the Pickering emulsion was blended, likely due to the enhanced hydrogen bonding interactions between alginate and the Pickering emulsion, which may strengthen the molecular network through hydrogen bonds. The absence of more distinct characteristic peaks for essential oil and CNC may be due to the overlap of these bands with the characteristic peaks of alginate. A similar observation was made by Wardana et al. [66] in their study of alginate/lemongrass oil/cellulose nanofibers for edible film/coating applications.

### 3.4. Antimicrobial Activity of Pickering Emulsion Thyme Essential Oil (PE–TEO) Against Several Microorganisms

The results of antimicrobial activity of TEO and its PE–TEO are shown in Table 3. The effectiveness of TEO and PE–TEO varied substantially, showing MIC values ranging from 1024 to 8192 µg/mL. TEO has an active antimicrobial activity, showing the strongest inhibition activity at MIC_50_ of 1024 µg/mL against *S. aureus* NBRC 13276. Moreover, TEO shown growth-inhibition activity against *S. aureus* ATCC 29213, *C. albicans* ATCC 14053, and *B. subtilis* NBRC 3009, at a respective MIC_50_ of 2048 µg/mL. Interestingly, while TEO did not show any inhibition activity against all tested microorganisms at MIC_80_ of >8192 µg/mL, the PE–TEO 10% showed an antimicrobial effect against *B. cereus* IFO 3001, *E. coli* NBRC 13500, *C. albicans* ATCC 14053, and *P. digitatum* NBRC 7758 at MIC_80_ of 8192 µg/mL, respectively. PE–TEO 10% also revealed its 50% of growth-inhibitory activity against *S. aureus* NBRC 13276, *E. coli* NBRC 13500, and *P. digitatum* NBRC 7758 at the respective MIC_50_ of 4096 µg/mL. PE–TEO 5% did not show any inhibitory activity against any tested microorganism (MICs > 8192 µg/mL).

Our results are in correspondence with the previous study, which revealed the antimicrobial activity of thyme white essential oil against *S. aureus* and *E. coli* at MIC of 0.625 µL/mL, respectively [31]. TEO has shown bacterial-inhibition activity against *S. aureus*, *E. coli*, and *C. albicans* at MIC of 0.125 mg/mL, 0.20 mg/mL, and 0.25 mg/mL, respectively [69]. Another study also reported the growth-inhibitory effect of TEO against *S. aureus*, *C. albicans*, and *B. subtilis* at MIC_90_ of 19.26 µL/mL, 159.26 µL/mL, and 16.56 µL/mL [70]. Antifungal activity of red TEO has been reported to inhibit the growth of *P. digitatum* at MIC of 66.6 µL/L, which indirectly explains the same effect found in this study. It is also reported the Pickering emulsion stabilized by CNC of thyme white essential oil exhibited antimicrobial activity against *S. aureus* at respective MIC of 0.25 µL/mL and *E. coli* at respective MIC of 0.312 µL/mL [31]. Another study reported the MIC_50_ of thyme nano emulsion against *E. coli* (62.5 mg/mL), *B. cereus* (250 mg/mL), and *C. albicans* (50 mg/mL) [71], which is higher than our findings. Despite numerous studies reporting the antimicrobial activity of TEO in emulsion form, to the best of our knowledge, this is the first study that reported the antifungal activity of CNC-stabilized PE–TEO against *P. digitatum*.

Pickering emulsion of essential oil has been reported to exhibit stronger antibacterial activity due to less prone to evaporation and oxidation [72], which corresponds to better inhibition activity of PE–TEO in this study. Pickering emulsions often exhibit better antimicrobial activity compared to traditional emulsions because the solid particles that stabilize the droplets form a protective barrier. This barrier enables a controlled release of antimicrobial agents, such as EOs, resulting in enhanced and prolonged antibacterial effects [73]. Commonly, a higher concentration of oil results in more oil droplets and a larger interfacial area that requires stabilization by Pickering particles, which can reduce the stability [60]. However, in this research, a different phenomenon was observed, where PE–TEO 10% exhibited better antimicrobial activity than the PE–TEO 5% formulation, likely due to the increased oil concentration enhancing CNC’s efficiency as a stabilizer in Pickering emulsions. One possible mechanism is that a higher TEO concentration leads to a more densely packed layer of CNC particles around each droplet, strengthening the mechanical barrier and preventing droplet coalescence [74]. Additionally, the extra oil droplets can promote a gel-like network within the emulsion, increasing viscosity and structural stability, thereby reducing the likelihood of phase separation [75], contributing to the better antimicrobial efficacy of PE–TEO.

Generally, the variations between this study and other findings on the antimicrobial activity of the TEO and PE–TEO tested could be due to multiple factors. It is important to consider the internal factors and external factors that could influence the amount of bioactive compound responsible for antimicrobial activity of TEO such as thymol and carvacrol. Moreover, different techniques for making emulsion, different antimicrobial testing methods, and different strains used, could also contribute to these discrepancies [76].

## 4. Conclusions

The results of this study clearly showed that using Pickering emulsions stabilized with cellulose nanocrystals (CNC) is an effective way to encapsulate thyme essential oil, enhancing both its stability and antimicrobial properties. These CNC-stabilized Pickering emulsions remained stable for over 21 days, outperforming traditional surfactant-based emulsions. Notably, the 10% TEO Pickering emulsion showed strong antimicrobial activity against various foodborne pathogens at MIC ranging from 4024 to 8192 µg/mL, suggesting it could be a promising natural preservative for food packaging and safety. Moving forward, further research should focus on refining the emulsion formulations for wider food preservation uses and testing them against a broader range of microbes to fully unlock the potential of Pickering emulsion of thyme essential oil (PE–TEO) for food preservation.

## Figures and Tables

**Table 1:** Chemical composition of TEO.

Peak	RT (Min)	RI	Relative Content (%)	Components	CAS Number
1	5.07	832	0.19	2-Hexenal	000505-57-7
2	5.75	-	0.06	Furan, 2,5-diethyltetrahydro-	041239-48-9
3	6.27		0.79	Bicyclo[3.1.0]hex-2-ene, 4-methyl-1-(1-methylethyl)-	028634-89-1
4	6.41	939	1.42	.alpha.-Pinene	000080-56-8
5	6.57	-	0.06	Bicyclo[3.1.0]hex-2-ene, 4-methylene-1-(1-methylethyl)-	036262-09-6
6	6.70	-	0.73	Bicyclo[2.2.1]heptane, 2,2-dimethyl-3-methylene-, (1S)-	005794-04-7
7	7.16	-	0.78	Bicyclo[3.1.1]heptane, 6,6-dimethyl-2-methylene-, (1S)-	018172-67-3
8	7.29	-	1.30	.beta.-Myrcene	000123-35-3
9	7.35	-	0.08	2,3-Dehydro-1,8-cineole	092760-25-3
10	7.41	994	0.27	3-Octanol	000589-98-0
11	7.62	1007	0.26	.alpha.-Phellandrene	000099-83-2
12	7.65	-	0.12	(+)-3-Carene	000498-15-7
13	7.79	1017	2.24	1,3-Cyclohexadiene, 1-methyl-4-(1-methylethyl)-	000099-86-5
14	7.94	1021	14.80	p-Cymene	000099-87-6
15	8.06	1033	3.74	Eucalyptol	000080-56-8
16	8.23	1050	0.18	1,3,6-Octatriene, 3,7-dimethyl-, (Z)-	003338-55-4
17	8.47	1064	6.07	γ.-Terpinene	000099-85-4
18	8.67	-	0.20	Cyclohexanol, 1-methyl-4-(1-methylethenyl)-, trans-	007299-40-3
19	8.90	1097	0.26	Cyclohexene, 1-methyl-4-(1-methylethylidene)-	000586-62-9
20	8.99	1095	0.22	Benzene, 1-methyl-4-(1-methylethenyl)-	001195-32-0
21	9.09	1098	1.69	Linalool	000078-70-6
22	9.54	1122	0.19	2-Cyclohexen-1-ol, 1-methyl-4-(1-methylethyl)-, trans-	029803-81-4
23	9.71	-	0.09	p-Mentha-1,5,8-triene	021195-59-5
24	9.83	1140	0.25	Bicyclo[3.1.1]heptan-3-ol, 6,6-dimethyl-2-methylene-, [1S-(1.alpha.,3.alpha.,5.alpha.)]-	000547-61-5
25	9.95	-	0.32	Ethanone, 1-(1,4-dimethyl-3-cyclohexen-1-yl)-	043219-68-7
26	10.05	1166	0.18	L-Menthone	014073-97-3
27	10.33	1169	4.13	endo-Borneol	000507-70-0
28	10.43	1177	2.82	Terpinen-4-ol	000562-74-3
29	10.58	1183	0.39	Benzenemethanol, .alpha.,.alpha.,4-trimethyl-	001197-01-9
30	10.67	1196	2.91	Estragole	000140-67-0
31	10.73		0.88	3-Cyclohexene-1-methanol, .alpha.,.alpha.,4-trimethyl-, (R)-	007785-53-7
32	11.13	-	0.36	cis-Chrysanthenyl formate	241123-18-2
33	11.22	1245	0.64	Benzene, 2-methoxy-1-methyl-4-(1-methylethyl)-	006379-73-3
34	11.42	1240	0.11	Benzaldehyde, 4-(1-methylethyl)-	000122-03-2
35	11.55		2.78	D-Carvone	002244-16-8
36	11.96	1289	7.46	Carvacrol	000499-75-2
37	12.14	1290	29.10	Thymol	000875-85-4
38	12.54	-	0.43	o-Isopropylphenetole	056631-59-5
39	12.87	-	0.13	Phenol, 2-methoxy-3-(2-propenyl)-	001941-12-4
40	13.24	1372	0.10	Copaene	003856-25-5
41	13.36	1384	0.16	(-)-.beta.-Bourbonene	005208-59-3
42	13.45	1401	0.15	Methyleugenol	000093-15-2
43	13.85	1418	0.50	Caryophyllene	000087-44-5
44	13.94	-	0.16	1,3,6,10-Dodecatetraene, 3,7,11-trimethyl-, (Z,E)-	026560-14-5
45	14.07	1455	0.43	5,9-Undecadien-2-one, 6,10-dimethyl-, (E)-	003796-70-1
46	14.32	-	0.07	1,4,7,-Cycloundecatriene, 1,5,9,9-tetramethyl-, Z,Z,Z-	1000062-61-9
47	14.51	1477	0.34	.gamma.-Muurolene	030021-74-0
48	14.75	1495	0.22	Naphthalene, 1,2,3,5,6,7,8,8a-octahydro-1,8a-dimethyl-7-(1-methylethenyl)-, [1R-(1.alpha.,7.beta.,8a.alpha.)]-	004630-07-3
49	14.88	1506	3.04	.beta.-Bisabolene	000495-61-4
50	15.04	-	0.59	1-Isopropyl-4,7-dimethyl-1,2,3,5,6,8a-hexahydronaphthalene	016729-01-4
51	15.10	-	0.14	4-isopropyl-1,6-dimethyl-1,2,3,4-tetrahydronaphthalene	1000378-99-6
52	15.27	1540	0.12	Cyclohexene, 4-[(1E)-1,5-dimethyl-1,4-hexadien-1-yl]-1-methyl-	025532-79-0
53	15.35	1546	0.07	.alpha.-Calacorene	021391-99-1
54	15.82	1571	0.45	1H-Cycloprop[e]azulen-7-ol, decahydro-1,1,7-trimethyl-4-methylene-, [1ar-(1a.alpha.,4a.alpha.,7.beta.,7a.beta.,7b.alpha.)]-	006750-60-3
55	15.91	1578	0.61	Caryophyllene oxide	001139-30-6
56	16.05	-	0.10	1H-Cyclopropa[a]naphthalene, 1a,2,3,5,6,7,7a,7b-octahydro-1,1,7,7a-tetramethyl-, [1aR-(1a.alpha.,7.alpha.,7a.alpha.,7b.alpha.)]-	017334-55-3
57	16.55	-	0.26	Bicyclo[4.4.0]dec-1-ene, 2-isopropyl-5-methyl-9-methylene-	150320-52-8
58	16.72	1653	0.16	ɑ-Cadinol	000481-34-5
Total compound identified (%)			96.32		

Notes: RT: Retention Times; RI: Retention Indices.

**Table 2:** Characteristics of PE–TEO and surfactant TEO.

Samples	Particle Size (nm)	Zeta Potential (mV)	Polydispersity Index
A	8917	−40.29	0.3522
B	4994	−38.91	0.2562
C	1682	−19.17	0.5548
D	619	−13.34	1.0000

Notes: A: CNC 0.2% + TEO 5% + Na-alginate 1%; B: CNC 0.2% + TEO 10% + Na-alginate 1%; C: Tween 80 1% + TEO 5% + Na-alginate; D: Tween 80 1%, TEO 10%, Na-alginate.

**Table 3:** Minimum inhibitory concentration (MIC) of TEO and PE–TEO.

Microorganisms	TEO		PE–TEO 5%		PE–TEO 10%		Tetracycline		Tioconazole	
	MIC_80_	MIC_50_	MIC_80_	MIC_50_	MIC_80_	MIC_50_	MIC_80_	MIC_50_	MIC_80_	MIC_50_
*Staphylococcus aureus* ATCC 29213	>8192	2048	>8192	>8192	>8192	8192	>8	1	-	-
*Staphylococcus aureus* ATCC 25923	>8192	4096	>8192	>8192	>8192	8192	>8	0.5	-	-
*Staphylococcus aureus* NBRC 13276	>8192	1024	>8192	>8192	>8192	4096	>32	1	-	-
*Bacillus subtilis* NBRC 3009	>8192	2048	>8192	>8192	>8192	>8192	>32	0.25	-	-
*Bacillus cereus* IFO 3001	>8192	4096	>8192	>8192	8192	8192	>32	0.5	-	-
*Escherichia coli* NBRC 13500	>8192	4096	>8192	>8192	8192	4096	>32	1	-	-
*Candida albicans* ATCC 14053	>8192	2048	>8192	>8192	>8192	8192	>32	1	-	-
*Penicillium digitatum* NBRC 7758	>8192	4096	>8192	>8192	8192	4096	-	-	>32	>32

## Data Availability

The original contributions presented in the study are included in the article. Further inquiries can be directed to the corresponding author.
